# Photosynthetic Traits of *Quercus coccifera* Green Fruits: A Comparison with Corresponding Leaves during Mediterranean Summer

**DOI:** 10.3390/plants13202867

**Published:** 2024-10-14

**Authors:** Dimitrios Kalachanis, Christos Chondrogiannis, Yiola Petropoulou

**Affiliations:** 1 Laboratory of Plant Physiology, Department of Biology, University of Patras, 26504 Patras, Greece; dimikalahan@gmail.com; 2 School of Natural Sciences, Botany, Trinity College Dublin, D02 PN40 Dublin, Ireland; chondroc@tcd.ie

**Keywords:** fruit photosynthesis, CO_2_ re-assimilation, drought stress, electron transport rate, non-photochemical quenching, photoinhibition, *Quercus coccifera* L.

## Abstract

Fruit photosynthesis occurs in an internal microenvironment seldom encountered by a leaf (hypoxic and extremely CO_2_-enriched) due to its metabolic and anatomical features. In this study, the anatomical and photosynthetic traits of fully exposed green fruits of *Quercus coccifera* L. were assessed during the period of fruit production (summer) and compared to their leaf counterparts. Our results indicate that leaf photosynthesis, transpiration and stomatal conductance drastically reduced during the summer drought, while they recovered significantly after the autumnal rainfalls. In acorns, gas exchange with the surrounding atmosphere is hindered by the complete absence of stomata; hence, credible CO_2_ uptake measurements could not be applied in the field. The linear electron transport rates (ETRs) in ambient air were similar in intact leaves and pericarps (i.e., when the physiological internal atmosphere of each tissue is maintained), while the leaf NPQ was significantly higher, indicating enhanced needs for harmless energy dissipation. The ETR measurements performed on leaf and pericarp discs at different CO_2_/O_2_ partial pressures in the supplied air mixture revealed that pericarps displayed significantly lower values at ambient gas levels, yet they increased by ~45% under high CO_2_/O_2_ ratios (i.e., at gas concentrations simulating the fruit’s interior). Concomitantly, NPQ declined gradually in both tissues as the CO_2_/O_2_ ratio increased, yet the decrease was more pronounced in pericarps. Furthermore, net CO_2_ assimilation rates for both leaf and pericarp segments were low in ambient air and increased almost equally at high CO_2_, while pericarps exhibited significantly higher respiration. It is suggested that during summer, when leaves suffer from photoinhibition, acorns could contribute to the overall carbon balance, through the re-assimilation of respiratory CO_2_, thereby reducing the reproductive cost.

## 1. Introduction

Leaves are considered the main photosynthetic organs of a plant, optimized for both efficient light absorption and exchange of interfering gases with the surrounding atmosphere. Yet, other largely heterotrophic plant parts, such as green flowers, fruits, petioles and stems, mainly fulfilling different primary functions, have been found to be photosynthetically active. Even light-remote tissues, like pith, xylem rays, deeply located seeds and roots, may contain functional chloroplasts [1,2,3,4,5,6,7,8,9,10].

We may distinguish between non-foliar plant structures characterized by an abundance of functional stomata on their epidermis (though fewer than the corresponding leaves, i.e., green stems and sterile flower parts) and those with a very low number or even a total absence of stomata (such as green fruits and peridermal twigs). In the first case, a net atmospheric CO_2_ uptake occurs, and photosynthesis resembles mostly that of the mesophyll [6,11]. In the latter, however, extremely high internal CO_2_ concentrations are induced, while O_2_ falls to very low or even hypoxic levels due to the hindered gas exchange with the surrounding atmosphere and the enhanced metabolic demands of these organs [1,5,12,13]. Thus, under such internal aerial conditions, completely different from that of leaves, photosynthesis is considered to serve the re-assimilation of respiratory CO_2_, contributing to the net carbon gain of the whole plant [1,5,14,15]. Alternatively, or in addition, another role assigned to the photosynthesis of these bulky organs is the protection against the cytoplasm’s acidification caused by high levels of CO_2_, and the alleviation of the negative effects of hypoxia [5,10,13,16].

Concerning green fruits, previous investigations have indicated that they exhibit very low CO_2_ assimilation rates compared to corresponding leaves [1,6,17,18]. Moreover, based on chlorophyll fluorescence measurements, fruits display similar or slightly lower photon-trapping efficiency (Fv/Fm) than leaves but considerably lower effective PSII yields (Φ_PSII_) and linear electron transport rates (ETRs), a rather large antenna size combined with an increased number of closed (i.e., non-QA-reducing) PSII centers and higher non-photochemical quenching (NPQ) [16,17,18,19,20,21]. According to earlier studies on four species with different fruit types (*Acacia cyanophylla*, *Ailanthus altissima*, *Nerium oleander* and *Rosa* sp.), the suppressed linear e^−^ flow of fruits was accompanied by a faster reduction of PSI final electron acceptors and a significantly higher potential for cyclic electron flow around PSI (CEF). This, in turn, may serve both to replenish the ATP lost due to hypoxia and develop an adequate NPQ through the generation of a high ΔpH [16,22]. 

Regarding photosynthetic pigments, green fruits possess lower contents of total chlorophylls (Chls) and carotenoids (Car) than leaves, a consistently higher ratio of total carotenoids to total chlorophylls (Car/Chls) and, usually, a lower Chl a/b ratio [6,16,17,18,22,23]. Judging from relevant studies on leaves, a low Chl a/b ratio is a typical characteristic of shade acclimation, reflecting a higher investment in light-harvesting complexes relative to reaction centers [24,25,26]. On the other hand, an increased Car/Chl ratio may denote either higher needs for photon capture [27] or increased demands for thermal dissipation of excess excitation energy [28,29]. Since growth irradiance, however, more strongly affects the levels of the potentially photoprotective than the photo-selective carotenoids [26,30,31], the latter interpretation seems more plausible and consistent with the higher NPQ values observed in fruits of various species [16,17,18,19,23]. Moreover, it was shown that the higher Car/Chl ratio in fruits is mainly shaped by the increased pools of photoprotective carotenoids (especially VAZ cycle components), accompanied by higher mid-day de-epoxidation (DEPS) values, which could serve their higher thermal dissipation needs [22,23,32].

The abovementioned features of fruit chlorenchyma also characterize photosynthesis in peridermal twigs, which takes place under analogous gas exchange restrictions [8,33,34,35,36,37,38]. Accordingly, it was suggested that such green bulky organs facing similar micro-environmental constraints may have adopted a common photosynthetic pattern to alleviate the negative effects of hypoxia and high CO_2_ concentrations in their interior [16,22].

In line with the abovementioned information on fruit photosynthesis, most of the published research focuses on edible species of commercial interest, mainly on crop yield improvement [1,6,10]. Since fruit chloroplasts operate in a peculiar internal microenvironment completely different from that of leaves, apart from studying the possible role(s) of fruit photosynthesis per se, they also constitute a useful system for investigating the adaptations and flexibility of the photosynthetic machinery in terms of the photosystem function and the processing of the absorbed light energy and electron flow. However, studies comparing photosynthetic traits between green fruits and their leaf counterparts are still limited in the literature, especially those designed to conduct such comparisons under physiological (for each tissue) internal gas ratios. The contribution of fruit photosynthesis to the overall carbon balance and reproductive cost of a plant is typically estimated using gas exchange or ^14^C-uptake techniques and, more recently, by measuring the in vivo Chl fluorescence (mainly by PAM fluorometry). Chlorophyll fluorescence measurements have been considered an advantageous tool for the evaluation of photosynthetic traits in intact fruits, both because they can detect photosynthetic activity in cases where the gas exchange method fails and because analysis of the individual fluorescence parameters provides information on the distribution of absorbed radiation in photochemical and non-photochemical quenching (on light-adapted samples) and on potential limitations of e^−^ flux and energy conversion through their photosystems (on dark-adapted samples) [1,10,16]. Yet, studies including comparisons of the fast chlorophyll a fluorescence transients (OJIP analysis) between leaves and fruits are scarce. Furthermore, at least to our knowledge, there are no such comparisons for any *Quercus* species.

Based on the above, we proceeded to the determination of the photosynthetic traits of *Quercus coccifera* green fruits using gas exchange and various chlorophyll fluorescence methods under different partial pressures of the interfering gases, simulating those of the inner atmosphere in the pericarp. Measurements were conducted during the period of green fruit production (summer), and the corresponding mature leaves served as controls. The anatomy of acorns was also evaluated as their morphological characteristics (such as stomatal density, cuticle thickness) are expected to influence/shape the light and gas levels in the fruit’s interior and, consequently, their photosynthetic activity.

## 2. Results

### 2.1. Anatomy

**Figure 1:**
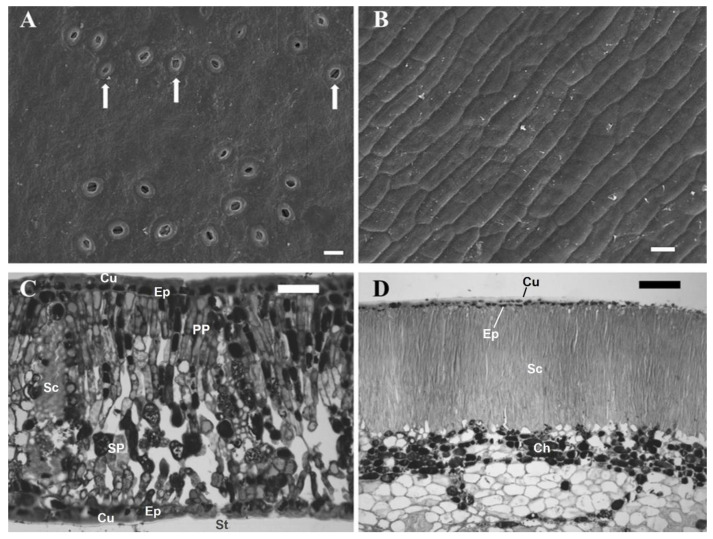
Fine structure of *Q. coccifera* leaves (left column) and pericarps (right column), as revealed by scanning electron microscope images (**A**,**B**) and light micrographs of cross sections (**C**,**D**). Stomata are indicated by arrows in the abaxial leaf surface (**A**), whereas no stomata could be found in pericarps (**B**). Samples were collected in August. In Figure 1A,B, bars = 20 μm; in Figure 1C,D. bars = 50 μm. Ch: chlorenchyma, Cu: cuticle, Ep: epidermis, PP: palisade parenchyma, SP: spongy parenchyma, Sc: sclerenchyma, St: stoma.

Leaves of *Quercus coccifera* display the typical anatomy of Mediterranean sclerophylls. Stomata are present only in the abaxial epidermis (Figure 1A), and a thick cuticle covers both leaf surfaces. Three rows of dense palisade parenchyma are found beneath the adaxial epidermis, whereas the spongy parenchyma with relatively small intercellular spaces lies above the abaxial one. In addition, as heterobaric leaves, they possess vertically oriented sclerenchymatous layers that divide the mesophyll into separate compartments (Figure 1C).

**Figure 2:**
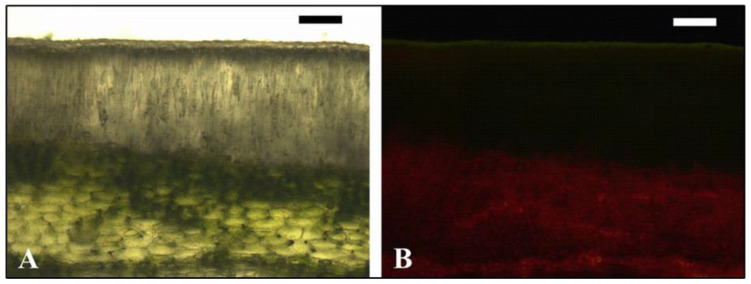
Light (**A**) and epifluorescence (**B**) microscope images of pericarp cross sections. Pericarps were collected in August. Bars = 100 μm.

The surface of fruits (i.e., pericarps) is characterized by the total absence of stomata (Figure 1B), and consequently, gas exchange with the surrounding atmosphere is hindered. The exocarp consists of a single-layered epidermis, covered by a thick cuticle, while a compact layer of sclerenchyma cells is located just below it. Beneath them lies the dense mesocarp with small intercellular spaces and numerous inclusions with solid compounds (Figure 1D). Chlorenchyma cells possessing functional chloroplasts are found in the upper 5–10 cell layers of the mesocarp (Figure 2A,B).

### 2.2. Photosynthetic Pigments

As shown in Table 1, total chlorophylls (on a surface area basis) did not differ substantially between leaves and fruits, while the Chl a/b ratio was significantly lower (ca. 25%) in pericarps. Compared to leaves, the total carotenoid concentration was ca. 50% higher in pericarps, leading to a ~30% increase in the corresponding Car/Chls ratio. In addition, absorptance (A) values, needed for the calculation of ETRs (see below), did not differ between leaves and fruits.

### 2.3. Chlorophyll Fluorescence Measurements in the Dark-Adapted State

**Figure 3:**
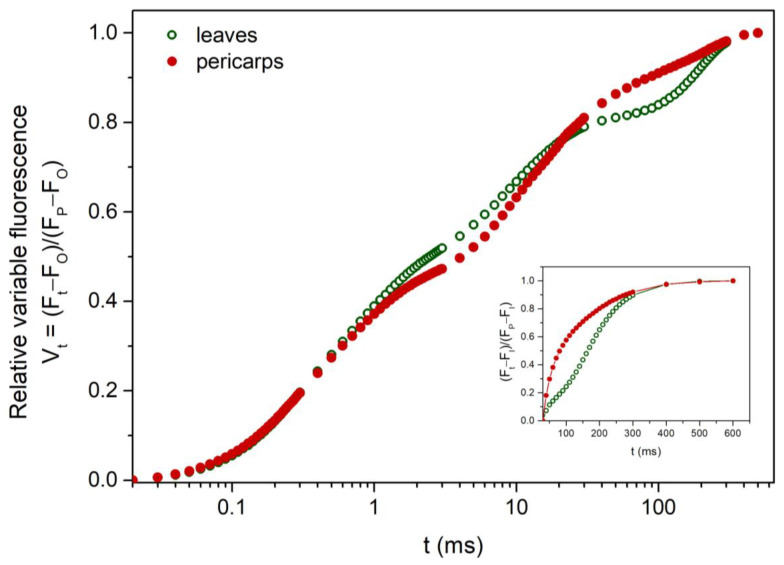
Fast chlorophyll *a* fluorescence transients (OJIP) from intact leaves (open green circles) and pericarps (closed red circles) in summer. Transients are given on a logarithmic time scale and are expressed as relative variable fluorescence (V_t_), i.e., after double normalization at the F_0_ and F_P_ steps. Insert shows the I-P part of the transient on a linear time scale, double normalized at the F_I_ and F_P_ steps. Each curve is the average of 30 independent transients.

Average fast chlorophyll fluorescence rise curves on a logarithmic time scale (OJIP transients) from leaves and fruits are presented in Figure 3. The transients, expressed as relative variable fluorescence (Vt), i.e., double-normalized between the two extreme fluorescence points O and P (at F_0_ and F_M_, respectively), were similar in both tissue types and displayed the typical polyphasic profile with distinct O-J-I-P steps. The main qualitative difference observed concerns the upper part of the curve (between I and P), which in pericarps lies higher than leaves; i.e., the relative I-P amplitude of pericarps was lower. Plotted on a linear time scale, after double normalization at the F_I_ and F_P_ steps, the I-P phase of the transient (i.e., between 30 ms and 600 ms) is better shown graphically in the insert of Figure 3. It is evident that the maximum fluorescence value is reached faster in pericarps, which in turn indicates a faster reduction of the PSI electron acceptor pools with electrons coming from the intersystem carriers. The rate constants of PSI reduction can be calculated as the time needed for the half saturation of these pools [t_1/2_^(I-P)^], which is almost half in pericarps (Table 2).

Table 2 presents biophysical parameters related to the structure and function of the photosynthetic machinery, derived after numerical analysis (JIP-test) of the fluorescence transients. The maximum yield of primary PSII photochemistry (φ_Po,_ equivalent to F_V_/F_M_) was high in both organs, with a slight trend for lower values in pericarps. The corresponding quantum yield (φ_Eo_) and efficiency (ψ_Eo_) of electron transport from reduced Q_A_ to intermediate electron carriers were similar in leaves and pericarps, while the efficiency of electron transfer from intermediate carriers to PSI terminal electron acceptors (δ_Ro_) and the quantum yield of reduction of the PSI electron acceptors (φ_Ro_) were slightly (~11%), yet significantly, lower in pericarps. In addition, the relative pool size of PSI final electron acceptors (1/V_I_), the content of PSI reaction centers (1-V_I_) and the relative measure of OEC inactivation (V_K_/V_J_) did not differ significantly. These results confirm the qualitative assessment of the chlorophyll fluorescence transients, namely the absence of significant limitations in linear electron flow at time zero (i.e., in dark-adapted samples) for both tissue types and a faster reduction of the PSI electron acceptors [as t_1/2_^(I-P)^] for pericarps. On the other hand, the specific fluxes of absorbed (ABS/RC), trapped (TR_O_/RC) and dissipated (DI_O_/RC) energy per active PSII (i.e., Q_A_-reducing) reaction center were increased by about 14%, 10% and 33%, respectively, in pericarps. The higher ABS/RC (which is indicative of the relative antenna size per active PSII reaction center) is in accordance with the lower Chl a/b ratio displayed by pericarps.

### 2.4. Chlorophyll Fluorescence Measurements in the Light-Adapted State

**Figure 4:**
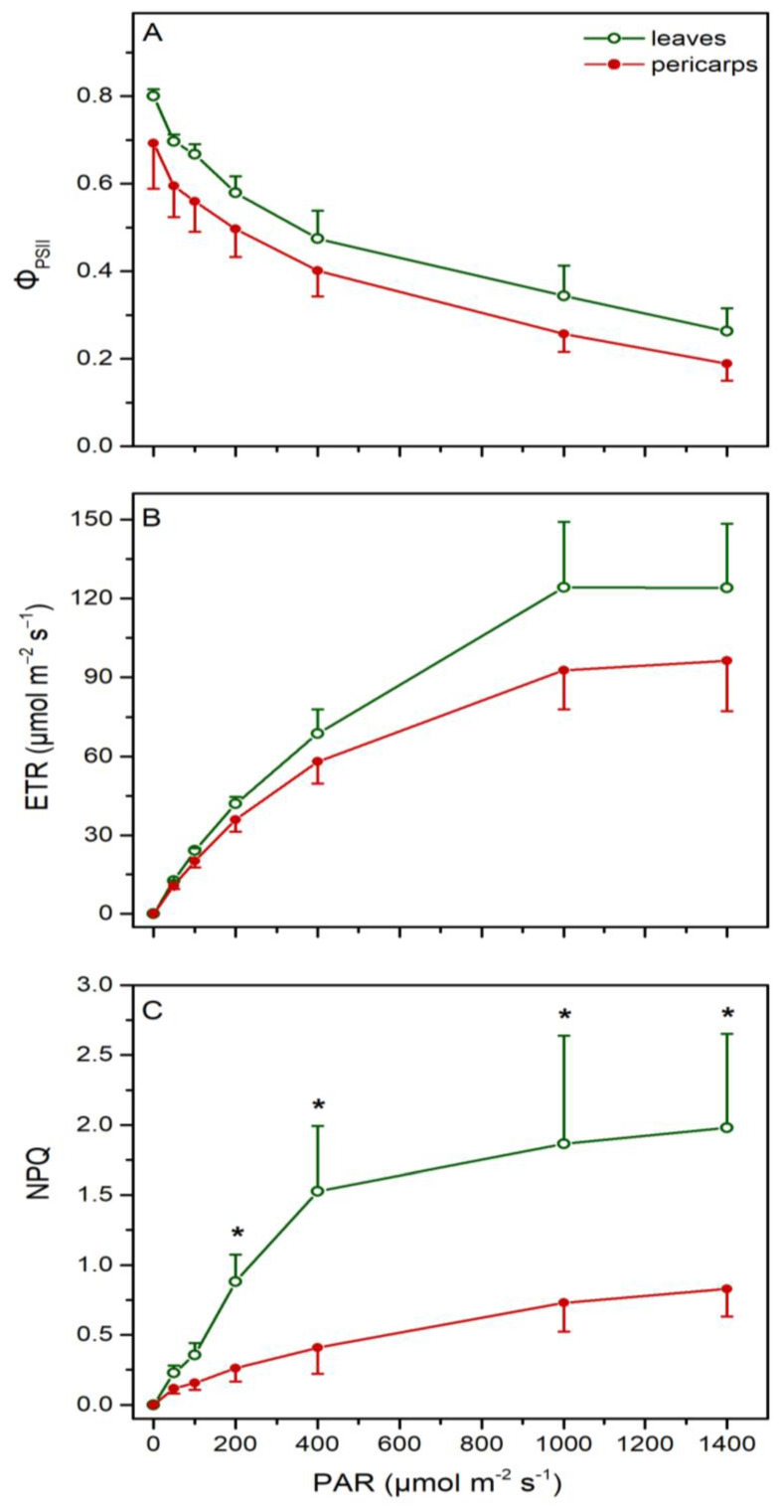
Light response curves of PSII quantum yield (Φ_PSII_, **A**), linear electron transport rate (ETR, **B**) and non-photochemical quenching (NPQ, **C**) from intact leaves (open green circles) and pericarps (closed red circles) in summer. Values are means ± SD from 6 independent measurements. Asterisks denote statistically significant differences (*p* < 0.05) between leaves and pericarps.

The light response curves of the effective PSII yield (Φ_PSII_) and linear electron transport rate (ETR) taken from intact leaves and fruits were similar, with a trend for lower (~17% on average) values in pericarps. Yet, leaves displayed higher values of non-photochemical quenching (NPQ) under all light levels tested (Figure 4).

**Figure 5:**
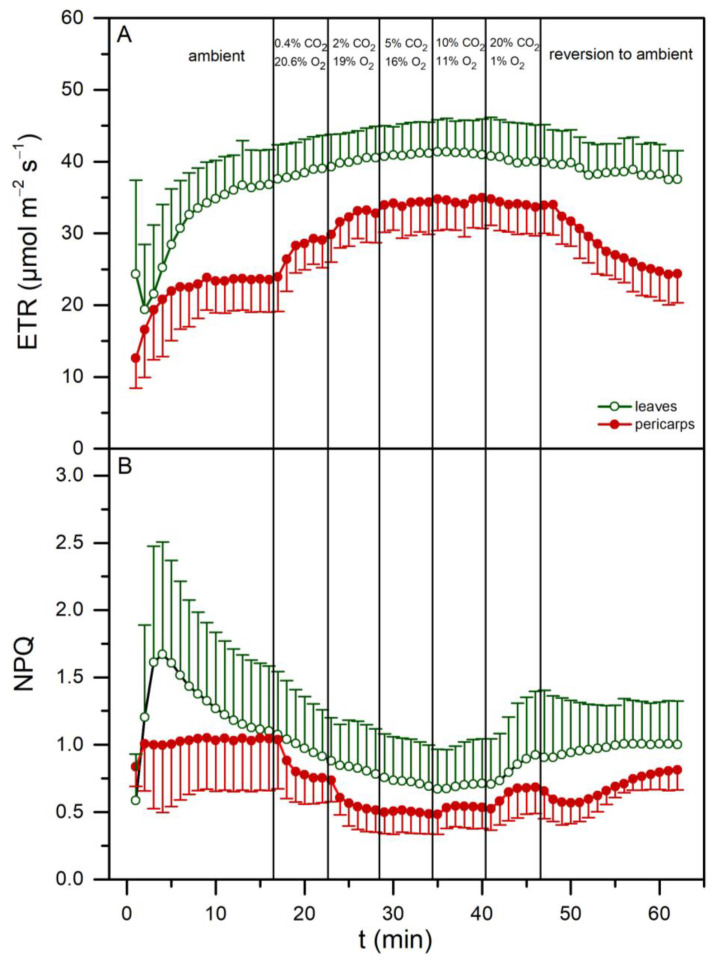
Induction curves of electron transport rate (ETR, **A**) and non-photochemical quenching (NPQ, **B**) at 200 μmol m^−2^ s^−1^ from leaf (open green circles) and pericarp (closed red circles) discs under ambient O_2_/CO_2_ concentrations. Subsequently, the samples were subjected to mutually varying external partial pressures of the interfering gases, i.e., a gradual CO_2_ increase and a concurrent O_2_ decrease, plus the reversion to ambient levels. Values are means ± SD from 8 independent measurements.

Induction curves of ETR and NPQ at 200 μmol m^−2^ s^−1^ from leaf and pericarp discs under ambient air conditions and their subsequent adjustment at mutually varying partial pressures of the interfering gases are presented in Figure 5. At ambient gas levels (0.04% CO_2_, 21% O_2_), the ETR of leaves was significantly higher than pericarps and increased slightly (~10%) at high CO_2_ and low O_2_ concentrations. On the other hand, the ETR of pericarps was substantially increased in the presence of elevated CO_2_/O_2_ ratios (Figure 5A, steps 3–5 after ambient air), i.e., under gaseous conditions simulating the acorn’s internal aerial environment, and, accordingly, the initially observed difference (~55%) between leaves and pericarps was diminished. Please note that the ETR values obtained from pericarp cut segments under high CO_2_/low O_2_ levels were comparable to those taken at the same irradiance from both intact leaves and fruits (see Figure 4B at 200 μmol m^−2^ s^−1^), i.e., when the ETR was measured under physiological internal gas concentrations for each organ. In addition, the recovery of ETR values after a return to the initial ambient air was almost full in both tissues, indicating that any effect of the intermediate gas mixtures was reversible (Figure 5A, last step). Furthermore, NPQ was gradually reduced in both tissue types under the increased CO_2_/O_2_ ratios, yet the decrease was more pronounced in pericarps (Figure 5B). When values from leaf and pericarp discs under physiological gas levels for each tissue were compared, the NPQ of leaves was more than 2-fold higher. Thus, the difference observed between intact leaves and acorns (see Figure 4C) was confirmed.

### 2.5. Gas Exchange Measurements

**Figure 6:**
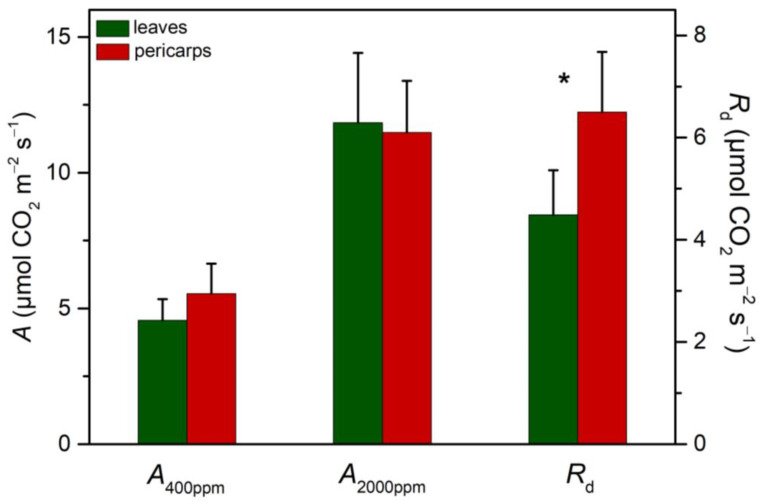
Net CO_2_ assimilation rate (*A*) at 200 μmol m^−2^ s^−1^, under 400 and 2000 ppm CO_2_ in the supplied air mixture, and dark respiration (*R*_d_, at 400 ppm CO_2_) from leaf and pericarp segments in summer. Values are means ± SD from 6 independent measurements. Asterisks denote statistically significant differences (*p* < 0.05) between leaves and pericarps.

The CO_2_ assimilation rates (*A*) at two different CO_2_ partial pressures (400 and 2000 ppm) of the externally supplied air mixture along with the dark respiration rate (*R*_d_, at 400 ppm) obtained from discs of leaves and pericarps are given in Figure 6. The CO_2_ concentrations used correspond to either normal (ambient) air or those simulating the internal gas levels of the pericarp, although 2000 ppm is rather an underestimation of the actual CO_2_ concentration expected in the fruit’s interior. Yet, due to the limitations of the injection system, we could not provide higher concentrations with the supplied gas mixture. *A* did not differ between leaves and pericarps at elevated CO_2_, while a trend, not statistically significant, for higher values (~20%) was observed in pericarps under ambient levels. In addition, *R*_d_ was significantly higher in pericarps than leaves, indicative of their higher metabolic needs.

**Figure 7:**
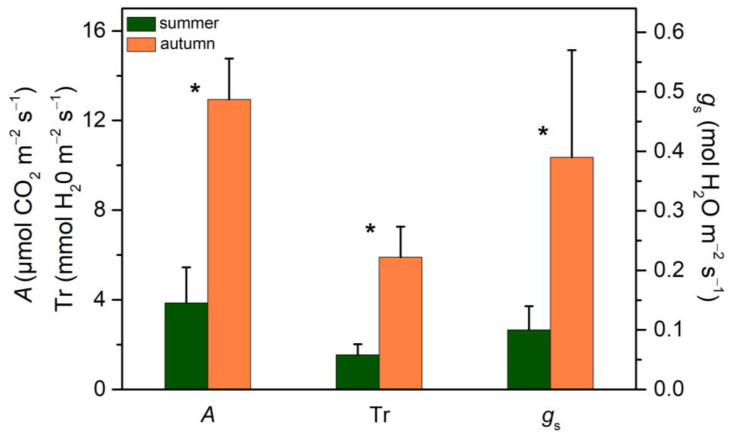
Net CO_2_ assimilation (*A*) and transpiration (T_r_) rates and stomatal conductance (*g*_s_) of leaves attached to the plant in August (green columns) and October (orange columns). PAR at 1420 μmol m^−2^ s^−1^. Values are means ± SD from 24 independent measurements. Asterisks denote statistically significant differences (*p* < 0.05) between summer and autumn for the indicated parameter.

In Figure 7, the net CO_2_ assimilation rate (*A*), transpiration rate (Tr) and stomatal conductance (*g*_s_) of leaves attached to the plant during summer (August) and in a favorable period (October) are presented. Reliable field CO_2_ uptake measurements on attached fruits could not be performed due to their absence of stomata. It is evident that in August, *A* and Tr are drastically reduced due to the significantly lower *g*_s_, while in October (after the autumnal rainfalls), all parameters recovered to normal values. Stomatal closure during the dry period reduces excessive water loss and thus protects the plant from dehydration. As a result, during the period of green fruit production, leaf net CO_2_ assimilation was very low.

## 3. Discussion

As mentioned in the Introduction, the present study aimed to evaluate the photosynthetic traits of acorns in comparison to their leaf counterparts, taking into account the prevailing CO_2_/O_2_ partial pressures in the internal microenvironment of the pericarps, which are seldom encountered by a leaf. All measurements were conducted in summer, i.e., during the period of green fruit production. To our knowledge, this is the first study carried out on *Q. coccifera* in this context.

It is evident from our results that fruits of *Q. coccifera*, apart from some similarities, also display characteristic deviations from the photosynthetic pattern previously reported for other plant species and fruit types. Firstly, total area-based chlorophylls of pericarps were as high as those of the corresponding leaves, while total carotenoids were significantly higher (~50%). At the same time, however, the relative Chl a/b ratio was lower while the Car/Chls ratio was higher in pericarps, in agreement with the previous investigations [6,16,17,18,22,23]. As it is known, light is essential for chlorophyll synthesis and photosynthetic activity and must penetrate the tissue’s interior to trigger the corresponding reactions [6,10]. Since the pericarp of *Q. coccifera* is covered by a thick cuticle, possibly limiting light penetration, the increased Car/Chl ratio may at first denote higher needs for light harvesting [27]. This is strengthened by the lower Chl a/b ratio of fruits pointing to a shade acclimation, as it reflects a greater size of light-harvesting antennas (containing both Chl a and Chl b) relative to reaction centers (containing only Chl a) [24,25]. In addition, the parameter ABS/RC evaluated through chlorophyll fluorescence was higher in pericarps, similarly indicating a larger antenna per active PSII reaction center. Yet, as it has been repeatedly shown in relevant studies on leaves, high area-based total chlorophyll and carotenoid concentrations correspond to sun-adapted rather than shade-adapted tissues [26,31]. Moreover, the sampled leaves and pericarps in our study were equally exposed to solar radiation, just to avoid any confounding effects imposed by their light history. As was shown in the leaves of *Phillyrea latifolia*, *Quercus coccifera* and *Vitis vinifera*, the transparent sclerenchymatous tissues (such as sclereids and bundle sheath extensions) may guide the light deep within the adjacent mesophyll areas, where photosynthetically active radiation is insufficient. In this way, the photosynthetic capacity of such heterobaric leaves could be improved [39,40]. An analogous light-guiding function could be inferred for the compact layer of sclereids surrounding the dense mesocarp of *Q. coccifera* fruits, thus triggering the conversion of proplastids into functional chloroplasts and chlorophyll synthesis. This is supported by the abundant chloroplasts located deep in the mesocarp cell layers and the strong red chlorophyll fluorescence they emit as well as the almost equal absorptance values observed in leaves and pericarps. Thus, as it has been suggested in previous studies, the corresponding pigment ratios (higher Car/Chl and lower Chl a/b) may be considered intrinsic attributes of fruit chlorenchyma related to other internal microenvironmental factors (e.g., hypoxia, extremely high CO_2_ concentrations) rather than shade acclimation [16,22]. 

A very important feature of *Q. coccifera* fruits is the complete absence of stomata along with the extremely small intercellular spaces in the mesocarp. In fruits devoid of stomata, gas exchange is attributed solely to cuticle involvement. Fruit cuticles are in most cases much less permeable than their leaf counterparts, depending on fruit type and developmental stage. In addition, they differ in their permeability for CO_2_ and O_2_, which is up to 10-fold higher for CO_2_ [1,6]. We may reasonably assume that these morphological features of acorns combined with their higher dark respiration rates create a steeper internal gradient of the interfering gases (i.e., extremely high CO_2_ concentrations and strong hypoxia) than that in fruits with even low stomatal density. A very low O_2_ partial pressure in the fruit’s interior limits the rate of oxidative phosphorylation [41], resulting in less ATP production. Concomitantly, as the pools of NADH are not oxidized, the Krebs cycle is inhibited due to NADH accumulation [42], and the ATP/NAD(P)H ratio is decreased.

Based on the above, one would expect that the electron transport constraints reported for fruits in previous studies (i.e., a suppressed linear electron flow along PSII in combination with enhanced CEF activity around PSI) would be more pronounced in *Q. coccifera* pericarps. In our case, however, the OJIP analysis of the chlorophyll fluorescence rise kinetics [43,44] showed no significant limitations in linear electron flow from PSII up to the intermediate e^−^ carriers (φ_Eo_, ψ_Eo_) for either leaves or pericarps. The slightly lower probabilities for electron transport from reduced intermediate carriers to PSI final acceptors (φ_Ro_, δ_Ro_) may indicate only moderate hindrance of linear electron flow along PSI [45,46], while the faster reduction of PSI electron acceptor pools [as t_1/2_^(I-P)^] points to an enhanced CEF activity in pericarps. Furthermore, since all the specific energy fluxes per active PSII reaction center (ABS/RC, TR_O_/RC, DI_O_/RC) were significantly higher in pericarps, an increased number of inactive PSII centers could be inferred [47]. As mentioned in the Introduction, an increased cyclic electron flow in pericarps would replenish the ATP lost due to hypoxia. We may argue, however, that an efficient linear flow (as in the case of *Q. coccifera* fruits) tends to increase the internal O_2_ concentration, thus alleviating, at least partly, the negative effects of hypoxia. Although CO_2_ is both the substrate and an activator of Rubisco, excessive CO_2_ concentrations could, however, inhibit photosynthesis due to the decrease in the stromal pH at suboptimal values. This effect is strengthened by low O_2_ levels [12]. On the other hand, as pointed out by Borisjuk and Rolletschek (2009), maintaining low O_2_ concentration in the fruit’s interior may be essential for normal seed development [13]. Judging from the above, an inherently enhanced CEF potential of pericarps, combined with a sufficient linear e^−^ flow, could act complementarily to restore the ATP/NADPH ratio and concurrently support the development of adequate NPQ through the generation of a high ΔpH. In any case, the differences in electron flow between leaves and fruits observed in the present study are of lower magnitude than those reported in our previous investigation [16].

The absence of noticeable limitations in the linear electron flow along PSII was confirmed by the fluorescence measurements on light-adapted material. In contrast to previous studies in a variety of species and fruit types [16,17,18,19,20,21], effective PSII yield (Φ_PSII_) and electron transport rate (ETR) measured under ambient gas levels were similar in leaves and pericarps at all light intensities tested, while non-photochemical quenching (NPQ) was significantly higher in leaves. Note that these measurements were performed on intact leaves and fruits, i.e., maintaining the physiological internal CO_2_ and O_2_ concentrations for each organ. When fluorescence measurements were conducted on leaf and pericarp discs at different CO_2_/O_2_ partial pressures in the externally supplied air mixture, it was shown that the ETR was significantly lower in pericarps at ambient gas levels and increased by 45% under high CO_2_/O_2_ ratios. In leaves, the ETR was much less responsive to gas changes, remaining almost constant throughout the applied air mixtures. Consequently, when ETR values obtained at realistic internal gas concentrations for each organ were compared, the initially observed difference between leaves and pericarps was substantially reduced, as they were similar to those measured in intact tissues at the same irradiance. Furthermore, NPQ assessed in leaf discs under ambient air was more than 2-fold higher compared to that of pericarps at elevated CO_2_/O_2_ ratios, in agreement with the findings obtained in intact leaves and fruits.

Taking the above information into account, we assessed the response of net CO_2_ assimilation rate in leaf and pericarp segments at two gas concentrations, one corresponding to normal air (400 ppm) and the other simulating the internal gas levels of the pericarp (2000 ppm), while O_2_ was kept constant at ambient values. Unfortunately, due to instrument limitations, it was not possible to exceed 2000 ppm in the supplied gas mixture, although the actual CO_2_ levels in pericarps are expected to be much higher. According to our results, both leaves and fruits displayed similar yet very low net CO_2_ uptake at 400 ppm, which increased to about the same extent at the elevated CO_2_ concentration. Concomitantly, the dark respiration rate in ambient air was significantly higher in pericarps. Such a high respiration rate is expected due to the increased metabolic demands of reproductive organs and contributes to an increase in partial CO_2_ pressure within pericarps [6,10,12].

A low net CO_2_ assimilation rate of leaves at ambient air combined with normal corresponding ETR values may indicate a diversion of electrons to alternative sinks (i.e., photorespiration). The protocol we used to measure ETRs at different gas partial pressures does not allow us such a discrimination, as potential photorespiration would be suppressed under the applied high CO_2_/O_2_ ratios. Note, however, that our measurements were carried out in summer (when *Q. coccifera* bears green fruits), which is considered particularly stressful for photosynthesis in the Mediterranean regions due to the coincidence of high irradiance, high temperature and low water availability [48]. Under drought stress, stomatal closure dominates to reduce excessive water loss, thus avoiding or delaying tissue dehydration [49]. Yet, carbon assimilation is restricted by the low CO_2_ availability, and the risk of photoinhibition is intensified [50,51]. In such cases, photorespiration is considered to function as an alternative sink of excess electrons [52,53]. Thus, leaves during the period of green fruit production were most likely photoinhibited, resulting in a very low net CO_2_ assimilation rate. On the other hand, in intact acorns, where the physiological internal CO_2_/O_2_ ratios are expected to be very high due to the absence of stomata and the elevated dark respiration rate, alternative electron paths are not favored [13,54]. Consequently, a significant surplus of CO_2_ is created within the pericarp, which the tissue could re-assimilate, thus contributing to the plant’s carbon balance and reproductive cost [6,10,14,15,55,56]. In this direction, CO_2_ assimilation rates increased substantially in both tissues under high CO_2_ levels. As we argued above, since ambient gas concentrations are quite normal for leaves but not for fruits, a comparison between leaves under ambient and fruits under high CO_2_ would be more appropriate.

To test the above considerations, net photosynthesis (*A*), transpiration (Tr) and stomatal conductance (g_s_) were measured in attached leaves in August and in October, after the autumnal rains. Not surprisingly, as stomatal conductance was 4-fold higher in autumn compared to summer, net photosynthesis increased more than 3 times, and transpiration was almost doubled. Thus, photoinhibition of leaves during summer was confirmed by our results, and, accordingly, they displayed a higher need for thermal dissipation of surplus excitation energy (NPQ). Although qE is considered the major component of NPQ, it may be also associated with photoinactivation processes (qI) and/or the redistribution of excitation energy between the photosystems (qT). As a result, the higher NPQ values in leaves than pericarps may not necessarily be associated with correspondingly enhanced Car/Chls ratios [57]. Photoinhibition of leaves during the summer months due to drought and high radiation levels has been observed in several species [58,59,60], including *Q. coccifera* [61,62]. On the other hand, the pericarp anatomy described above not only leads to high CO_2_ in the fruit’s interior, but may also reduce excessive water loss, thus increasing water-use efficiency. In this sense, the photosynthesis of reproductive organs (ROP) is often considered more resistant to abiotic stresses than that of their leaf counterparts [10].

Based on the results of the present study, we conclude that leaves of *Q. coccifera* most likely suffer from photoinhibition in summer. Although carbon re-assimilation in intact fruits could not be measured directly, judging from the photosynthetic behavior of pericarps, especially under different CO_2_/O_2_ partial pressures, we may reasonably argue that acorns could contribute to the overall carbon balance by efficiently refixing the respiratory CO_2_, thus reducing the reproductive cost during a stressful period for the leaves.

## 4. Materials and Methods

### 4.1. Plant Material, Experimental Site, and Sampling 

Green fruits (acorns) and the corresponding mature leaves from *Quercus cocciferra* L. (Fagaceae) individuals, growing wild in the vicinity of the Patras University Campus (38°14′ N, 21°44′ E, alt.125 m), were used throughout the study. *Q. coccifera* is an evergreen sclerophyllous Mediterranean shrub, characteristic of macchia vegetation, bearing fruits from mid-summer to mid-autumn. Sampling was performed always on clear days from late July to late September, when acorns are green and sufficiently uncovered from the cupulus (about two-thirds of the nut’s size). Field gas exchange measurements in attached leaves were conducted in August and mid-October. The climate of the sampling area is typically Mediterranean, with cool, wet winters and hot, dry summers [48]. During the experimental period, the mean monthly temperature ranged from 27.6 °C in July to 19.6 °C in October, while the monthly total precipitation was 0.2, 0.0, 13.4 and 141.2 mm for July, August, September and October, respectively.

On each sampling date, adequate numbers of intact fruits and leaves were harvested in the late afternoon, put in air-tight plastic envelopes containing moistened filter paper to avoid water losses, and kept all night in the dark at room temperature to be measured the next morning. Care was taken to harvest south-facing green fruits and leaves fully exposed to solar radiation to avoid any confounding effects of light or shade. In the case of fruits, the exposed side was labeled, and subsequently, all measurements were performed on this side. All measurements, except those of field gas exchange in attached leaves, were conducted under laboratory conditions.

### 4.2. Tissue Fixation for Light and Scanning Electron Microscopy

Leaf and pericarp samples were carefully cut and fixed in 5% glutaraldehyde in phosphate buffer (pH 7) for 2 h at room temperature. The tissue was then post-fixed in 1% OsO4 at 4 °C and dehydrated in a graded acetone series. For light microscopy, tissue samples were embedded in Durcupan ACM (Fluka, Buchs, Switzerland). Semi-thin sections (1–2 μm thick) of plastic embedded tissue made on a Reichert Om-U2 (Wien, Austria) microtome using glass knives were stained with Toluidine Blue O. The sections were examined with a Zeiss Axioplan microscope (Zeiss, Oberkochen, Germany) equipped with epi-fluorescence optics (HBO 50 W mercury lamp) and recorded using a digital camera (AxioCamMRc 5, Zeiss).

For scanning electron microscopy (SEM), dehydrated tissue samples were critical-point-dried, mounted with double adhesive tape on stubs, sputter-coated with gold and observed with a JEOL 6300 (JEOL, Tokyo, Japan) SEM microscope.

### 4.3. Fresh Plant Material and Epi-Fluorescence Microscopy

Cross sections, 40 μm thick, of pericarps were made using a Leica sliding microtome (Leica SM 2000 R, Nussloch, Germany). Chlorophyll autofluorescence was detected using a blue excitation filter set (Zeiss: exciter filter 450–490, chromatic beam splitter 510 and barrier filter LP520). The sections were examined and digitally recorded as above.

### 4.4. Photosynthetic Pigments

Discs of known area from pericarps and leaves were punched out and extracted with DMSO (dimethylsulfoxide) for 2 h at 65 °C [63] in the presence of a small amount of CaCO_3_ to avoid acidification and the concomitant pheophytinization of chlorophylls. The extract was centrifuged at 7.000 g for 10 min, and the clear supernatant was measured spectrophotometrically using a Shimadzu (UV-160A, Kyoto, Japan) double-beam spectrophotometer. The concentrations of chlorophyll a, chlorophyll b and total carotenoids were estimated according to the equations of Wellburn, 1994 [64]. Pigment ratios (Chl a/b and Car/Chls) were also estimated.

### 4.5. Chlorophyll Fluorescence Measurements in the Dark-Adapted Material

Intact, overnight-darkened leaves and fruits were used. All manipulations before fluorescence induction were performed under dim light of less than 0.5 μmol m^−2^ s^−1^, and samples were kept in the corresponding leaf clips for a further 30 min prior to measurements.

Fast chlorophyll *a* fluorescence transients were captured using a high-time-resolution fluorometer (Handy-PEA, Hansatech Instruments Ltd., King’s Lynn, Norfolk, UK). For excitation, a band of three red LEDs (peak at 650 nm) providing 3000 μmol m^−2^ s^−1^ at the sample level was used. Fluorescence was recorded from 10 μs to 2 s in the time intervals of 10–300 μs, 0.3–3 ms, 3–30 ms, 30–300 ms and 0.3–2 s, with a corresponding data acquisition rate of 10^5^, 10^4^, 10^3^, 10^2^ and 10 readings s^−1^, respectively (i.e., with time intervals of 10 μs, 100 μs, 1 ms, 10 ms and 100 ms between the readings). The following cardinal points in the fluorescence *vs.* time curve were used for the further calculation of biophysical parameters: the minimum fluorescence intensity at 50 μs (F_0_, when all RCs are open), the maximum fluorescence intensity (F_M_, when all RCs are closed), the fluorescence intensity at 300 μs (F_300μs_) needed for the calculation of the initial slope of the fluorescence transient (M_0_), and the fluorescence intensities at 2 ms (F_J_) and 30 ms (F_I_). From these primary data, the following parameters were derived according to the JIP-test [43], as extended to analyze events in or around PSI [44,45,46,65]:

(a) The quantum yields and efficiencies:

φ_Po_ = TR_0_/ABS = 1 − (F_0_/F_M_), the maximum quantum yield of primary photochemistry, equivalent to F_V_/F_M_ (where ABS and TR stand for the excitation energy absorbed and trapped by PSII);

ψ_Eo_ = ET_0_/TR_0_ = (F_M_ − F_J_)/(F_M_ − F_0_), the efficiency of the conservation of trapped excitation energy as electron transfer (ET) beyond Q_A_;

φ_Eo_ = ET_0_/ABS = 1 − (F_J_/F_M_), the quantum yield of electron transfer to intermediate electron carriers;

δ_Ro_ = RE_0_/ET_0_ = (F_M_ − F_I_)/(F_M_ − F_J_), the efficiency of electron transfer from intermediate carriers to the end electron acceptors of PSI (where RE denotes the reduction of PSI final electron acceptors);

φ_Ro_ = φ_Po_ × ψ_Eo_ × δ_Ro_ = 1 − (F_I_/F_M_), the quantum yield of reduction of PSI final electron acceptors.

(b) The specific fluxes per active (i.e., Q_A_-reducing) reaction center (RC):

ABS/RC = (M_0_/V_J_) × F_M_/(F_M_-F_0_) for absorption;

TR_0_/RC = M_0_/V_J_ for trapping;

DI_0_/RC = (M_0_/V_J_) × (F_0_/F_V_) for dissipation.

(c) 1/V_I_ = (F_M_ − F_0_)/(F_I_ − F_0_), the relative pool size of the final PSI electron acceptors;

1 − V_I_ = (F_M_ − F_I_)/(F_M_ − F_0_), the relative amplitude of the I–P phase reflecting the content of PSI reaction centers;

t_1/2_ ^(I-P)^, the half-rise time from F_I_ to F_P_ reflecting the time needed for half saturation of the final acceptors of the PSI pool with electrons donated by intermediate carriers;

V_K_/V_J_ = (F_300μs_ − F_0_)/(F_J_ − F_0_), the relative amplitude of the K band as a relative measure of oxygen-evolving complex (OEC) inactivation.

### 4.6. Chlorophyll Fluorescence Measurements in the Light-Adapted Material

Intact dark-adapted leaves and fruits were measured, and all manipulations were performed under dim light of less than 0.5 μmol m^−2^ s^−1^. Light curves were recorded with a Mini-PAM pulse-amplitude-modulated fluorometer (Walz, Effeltrich, Germany) equipped with a red LED source providing a weak measuring beam (<0.05 μmol m^−2^ s^−1^) plus a white halogen source providing saturation pulses (8000 μmol m^−2^ s^−1^, 0.8 s) and actinic light. Initially, the minimum (F_0_) and maximum (F_Μ_) fluorescence were obtained (before and during the saturation pulse, respectively) to calculate the maximum dark-adapted PSII yield as F_V_/F_M_ = (F_M_ − F_0_)/F_M_ [66]. Then, induction curves at 200 μmol m^−2^ s^−1^ were performed, and subsequently, samples were illuminated with stepwise increasing actinic irradiances from 0 to 1400 μmol m^−2^ s^−1^. Under each irradiance level, the steady-state fluorescence (F_s_) was recorded, and a saturating pulse was imposed (every 30 s) to determine the maximum light-adapted fluorescence level (F′_M_). Hence, light-adapted PSII yield (Φ_PSII_) was computed as Φ_PSII_ = (F′_M_ − F_s_)/F′_M_. The duration of each actinic step (typically not less than 4 min) was enough to obtain stable readings of PSII photochemical efficiency. Linear electron transport rate was computed as ETR = Φ_PSII_ × PAR × A × 0.5, where PAR is the incident photosynthetically active radiation, A the sample absorptance and 0.5 holds for the assumed equal distribution of absorbed energy between the two photosystems [66]. Non-photochemical energy quenching was computed as NPQ = (F_M_/F′_M_) – 1 = (F_M_ − F′_M_)/F′_M_ [67].

Leaf and pericarp absorptance values were obtained in separate measurements using an Imaging-PAM system (IMAG-MIN/B, Walz, Effeltrich, Germany) equipped with 12 blue LEDs (providing measuring light, actinic light and saturation pulses) and 20 red (R) and near-infrared (NIR) LEDs (peaks at 660 and 780 nm, respectively) for measuring PAR absorptivity and a CCD camera. This instrument probes the sample reflectance in the red (650 nm) and infrared (780 nm) band, and relative absorptance (A) is then estimated through a built-in equation as 1 − (R_650_/R_780_) [68], to be used for the calculation of the ETR (in the measurements with Mini-PAM). A white filter paper equally reflecting in the two spectral bands was used for system calibration. Leaf and pericarp segments were placed in Petri dishes on moistened filter paper, and the naturally exposed surface of plant material was probed. The signal used was integrated over the whole sample area, avoiding the edges which were unavoidably wounded by cutting [9,69,70].

### 4.7. Effects of Varying External Gas Partial Pressures on Light-Adapted Material

This set of measurements was performed to assess the linear electron transport rate (ETR) and non-photochemical quenching (NPQ) under varying gas mixtures simulating the internal aerial conditions of the pericarp. To this end, leaf and pericarp discs were put on moistened filter paper in a homemade, flow-through cuvette, which was adjusted to the leaf clip (leaf clip holder 2030-B) of a Mini-PAM for capturing fluorescence. The use of cut segments bypasses gas exchange limitations between plant tissues and the cuvette interior. All manipulations were performed under dim light of less than 0.5 μmol m^−2^ s^−1^. A G400 gas mixing pump (Qubit systems Inc., Kingston, ON, Canada) and pure gas cylinders of N_2_, O_2_ and CO_2_ (Air Liquide, Athens, Greece) were used to produce the desired gas mixture which, after passing through a humidifier, was led to the cuvette at a flow rate of 1 mL min^−1^. At the end of the experimental assembly, an air flowmeter was connected to check the flow [22]. The samples were illuminated at 200 μmol m^−2^ s^−1^ by an external white light source (halogen lamp 2050-HB). The ETR and NPQ were calculated as described above. Initially, ambient gas concentrations (i.e., 0.04% CO_2_, 21% O_2_, 78.96% N_2_) were pumped to the samples, and induction curves were performed. Subsequently, gas mixtures with gradually increasing CO_2_ and concurrently decreasing O_2_ partial pressures were supplied in the chamber (in five consecutive steps, plus recovery to ambient air), while N_2_ was kept constant. The duration of each step was enough to achieve equilibrium in the gas concentration between the chamber and the tissue’s interior, not less than 6 min in any case.

### 4.8. Gas Exchange Measurements

For gas exchange measurements, an open gas analyzer (Li-6400, Li-Cor, Lincoln, NE, USA) equipped with the standard Li-6400 leaf chamber, an LED light source (6400-02B, with both red and blue LEDs) and a CO_2_-regulating device (6400-01 CO_2_ Injector System) was used. To compare leaf and fruit photosynthetic rates, gas exchange measurements were carried out under constant temperature (27 °C) and relative humidity (75%). Net photosynthesis (*A*) was measured in leaf and pericarp segments under light intensity of 200 μmol m^−2^ s^−1^ and at 2 different CO_2_ concentrations (400 and 2000 ppm) in the supplied air mixture. The dark respiration rate (*R*_d_) was measured at 400 ppm after the corresponding plant tissues were darkened within the chamber for 30 min.

In addition, field measurements were conducted on intact (attached) leaves in August, i.e., during summer drought, and in October, after the autumnal rainfalls. Leaf maximum photosynthetic rate (*A*_max_), transpiration rate (Tr) and stomatal conductance (*g*_s_) were measured under field conditions (temperature was 35 and 25 °C in summer and autumn, respectively). Light intensity was saturating (1500 ± 100 μmol m^−2^ s^−1^), and the CO_2_ reference was set near the ambient concentration at 400 ppm.

### 4.9. Statistical Analysis

The significance of differences for the measured parameters between leaves and pericarps was assessed by one-way ANOVA (SPSS v.25.0 statistical package, IBM-SPSS Statistics, Armonk, NY, USA). The number of independent measurements in each case is given in the legends of figures and tables.

## Figures and Tables

**Table 1:** Surface-area-based total chlorophyll (Chl) and carotenoid (Car) content (μg cm^−2^), the corresponding pigment ratios and absorptance of leaves and pericarps during summer.

Pigments	Leaves	Pericarps
Chls	39.75 ± 3.59 a	44.69 ± 6.32 a
Car	8.46 ± 0.46 a	12.75 ± 1.24 b
Chl a/b	2.61 ± 0.13 a	1.95 ± 0.18 b
Car/Chls	0.21 ± 0.01 a	0.28 ± 0.02 b
Absorptance	0.724 ± 0.044 a	0.723 ± 0.046 a

Values are means ± SD from 15 independent measurements. Different letters within each row indicate statistically significant differences (*p* < 0.05) between leaves and pericarps for the indicated parameter.

**Table 2:** Numerical values of quantum yields and flux ratios (φ_Po_, φ_Eo_, ψ_Eo,_ φ_Ro_, δ_Ro_) and specific energy fluxes per Q_A_-reducing PSII center (ABS/RC, TR_0_/RC, DI_0_/RC) in leaves and pericarps during summer. Moreover, the ratios V_K_/V_J_ (as a relative measure of OEC inactivation), 1/V_I_ (as a relative measure of the pool size of PSI final electron acceptors), 1-V_I_ (as a relative measure of the content of PSI reaction centers) and the half-rise time from F_I_ to F_P_ (t_1/2_^(I-P^) are given. Definitions and formulae are given in Materials and Methods.

Parameter	Leaves	Pericarps
φ_Po_	0.81 ± 0.02 a	0.79 ± 0.04 b
φ_Eo_	0.44 ± 0.05 a	0.43 ± 0.05 a
ψ_Eo_	0.55 ± 0.05 a	0.57 ± 0.04 a
φ_Ro_	0.19 ± 0.03 a	0.17 ± 0.04 b
δ_Ro_	0.44 ± 0.05 a	0.39 ± 0.06 b
V_K_/V_J_	0.35 ± 0.05 a	0.38 ± 0.06 a
1/V_I_	1.32 ± 0.06 a	1.29 ± 0.06 a
1-V_I_	0.24 ± 0.03 a	0.22 ± 0.04 a
t_1/2_^(I-P)^ (msec)	136 ± 23 a	62 ± 24 b
ABS/RC	1.74 ± 0.26 a	1.99 ± 0.39 b
TR/RC	1.41 ± 0.19 a	1.55 ± 0.24 b
DI_o_/RC	0.33 ± 0.08 a	0.44 ± 0.16 b

Values are means ± SD from 30 independent measurements. For each parameter, different letters indicate statistically significant differences (*p* < 0.05) between leaves and pericarps.

## Data Availability

The data presented in this study are available in the figures and tables of the manuscript.

## References

[1] Blanke M.M., Lenz F. (1989). Fruit photosynthesis. Plant Cell Environ..

[2] Dogane Y., Ando T. (1990). An estimation of carbon evolution during flowering and capsule development in a *Laeliocattleya* orchid. Sci. Hortic..

[3] Nilsen E.T., Karpa D., Mooney H.A., Field C. (1993). Patterns of stem photosynthesis in two invasive legumes (*Spartium junceum*, *Cytisus scoparius*) of the California coastal region. Am. J. Bot..

[4] Clement C., Mischler P., Burrus M., Audran J.C. (1997). Characteristics of the photosynthetic apparatus and CO_2_-fixation in the flower bud of Lilium. I. Corolla. Int. J. Plant Sci..

[5] Pfanz H., Aschan G., Langefeld-Heyser R., Wittman C., Loose M. (2002). Ecology and ecophysiology of tree stems–corticular and wood photosynthesis. Naturwissenschaften.

[6] Aschan G., Pfanz H. (2003). Non-foliar photosynthesis—A strategy of additional carbon acquisition. Flora.

[7] Dima E., Manetas Y., Psaras G.K. (2006). Chlorophyll distribution pattern in inner stem tissues: Evidence from epifluorescence microscopy and reflectance measurements in 20 woody species. Trees.

[8] Yiotis C., Petropoulou Y., Manetas Y. (2009). Evidence for light-independent and steeply decreasing PSII efficiency along twig depth in four tree species. Photosynthetica.

[9] Yiotis C., Manetas Y. (2010). Sinks for photosynthetic electron flow in green petioles and pedicels of *Zantedeschia aethiopica*: Evidence for innately high photorespiration and cyclic electron flow rates. Planta.

[10] Brazel A.J., Ó’Maoiléidigh D.S. (2019). Photosynthetic activity of reproductive organs. J. Exp. Bot..

[11] Nilsen E.T., Gartner B. (1995). Stem photosynthesis: Extent, patterns, and role in plant carbon economy. Plant Stems: Physiology and Functional Morphology.

[12] Goffman F.D., Ruckle M., Ohlrogge J., Shachar-Hill Y. (2004). Carbon dioxide concentrations are very high in developing oilseeds. Plant Physiol. Biochem..

[13] Borisjuk L., Rolletschek H. (2009). The oxygen status of the developing seed. New Phytol..

[14] Bazzaz F.A., Carlson R.W., Harper J.L. (1979). Contribution to reproductive effort by photosynthesis of flowers and fruits. Nature.

[15] Carrara S., Pardossi A., Soldatini G.F., Tognoni F., Guidi L. (2001). Photosynthetic activity of ripening tomato fruit. Photosynthetica.

[16] Kalachanis D., Manetas Y. (2010). Analysis of fast chlorophyll fluorescence rise (O-K-J-I-P) curves in green fruits indicates electron flow limitations at the donor side of PSII and the acceptor sides of both photosystems. Physiol. Plant..

[17] Ranjan S., Singh R., Soni D.K., Pathre U.V., Shirke P.A. (2012). Photosynthetic performance of *Jatropha curcas* fruits. Plant Physiol. Biochem..

[18] Ferroni L., Pantaleoni L., Baldisserotto C., Aro E.M., Pancaldi S. (2013). Low photosynthetic activity is linked to changes in the organization of photosystem II in the fruit of *Arum italicum*. Plant Physiol. Biochem..

[19] Hetherington S.E., Smillie R.M., Davies W.J. (1998). Photosynthetic activities of vegetative and fruiting tissues of tomato. J. Exp. Bot..

[20] Lemos Filho J.P., Isaias R.M.S. (2004). Comparative stomatal conductance and chlorophyll a fluorescence in leaves vs. fruits of the cerrado legume tree, *Dalbergia miscolobium*. Braz. J. Plant Physiol..

[21] Aschan G., Pfanz H., Vodnik D., Batič F. (2005). Photosynthetic performance of vegetative and reproductive structures of green hellebore (*Helleborus viridis* L. agg.). Photosynthetica.

[22] Kyzeridou A., Stamatakis K., Petropoulou Y. (2015). The non-foliar hypoxic photosynthetic syndrome: Evidence or enhanced pools and functionality of xanthophyll cycle components and active cyclic electron flow in fruit chlorenchyma. Planta.

[23] Cheng L., Ma F. (2004). Diurnal operation of the xanthophyll cycle and the antioxidant system in apple peel. J. Am. Soc. Hortic. Sci..

[24] Anderson J.M. (1986). Photoregulation of the composition, function, and structure of thylakoid membranes. Annu. Rev. Plant Physiol..

[25] Murchie E.H., Horton P. (1998). Contrasting patterns of photosynthetic acclimation to the light environment are dependent on the differential expression of the responses to altered irradiance and spectral quality. Plant Cell Environ..

[26] Lichtenthaler H.K., Ač A., Marek M.V., Kalina J., Urban O. (2007). Differences in pigment composition, photosynthetic rates and chlorophyll fluorescence images of sun and shade leaves of four tree species. Plant Physiol. Biochem..

[27] Demmig-Adams B., Gilmore A.M., Adams W.W. (1996). In vivo functions of carotenoids in higher plants. FASEB.

[28] Demmig-Adams B., Adams W.W. (1996). The role of xanthophyll cycle carotenoids in the protection of photosynthesis. Trends Plant Sci..

[29] Choudhury N., Behera R. (2001). Photoinhibition of photosynthesis: Role of carotenoids in photoprotection of chloroplast constituents. Photosynthetica.

[30] Thayer S.S., Björkman O. (1990). Leaf xanthophyll content and composition in sun and shade determined by HPLC. Photosynth. Res..

[31] Demmig-Adams B. (1998). Survey of Thermal Energy Dissipation and Pigment Composition in Sun and Shade Leaves. Plant Cell Physiol..

[32] Esteban R., Olascoaga B., Becerril J.M., García-Plazaola J.I. (2010). Insights into carotenoid dynamics in non-foliar photosynthetic tissues of avocado. Physiol. Plant..

[33] Manetas Y. (2004). Probing corticular photosynthesis through in vivo chlorophyll fluorescence measurements: Evidence that high internal CO_2_ levels suppress electron flow and increase the risk of photoinhibition. Physiol. Plant..

[34] Levizou E., Petropoulou Y., Manetas Y. (2004). Carotenoid composition of peridermal twigs does not fully conform to a shade acclimation hypothesis. Photosynthetica.

[35] Kotakis C., Petropoulou Y., Stamatakis K., Yiotis C., Manetas Y. (2006). Evidence for active cyclic electron flow in twig chlorenchyma in the presence of an extremely deficient linear electron transport activity. Planta.

[36] Ivanov A.G., Krol M., Sveshnikov D., Malmberg G., Gardeström P., Hurry V., Öquist G., Huner N.P.A. (2006). Characterization of the photosynthetic apparatus in cortical bark chlorenchyma of Scots pine. Planta.

[37] Filippou M., Fasseas C., Karabourniotis G. (2007). Photosynthetic characteristics of olive tree (Olea europaea) bark. Tree Physiol..

[38] Levizou E., Manetas Y. (2008). Maximum and effective PSII yields in the cortex of the main stem of young *Prunus cerasus* trees: Effects of seasons and exposure. Trees.

[39] Karabourniotis G. (1998). Light-guiding function of foliar sclereids in the evergreen sclerophyll *Phillyrea latifolia*. J. Exp. Bot..

[40] Karabourniotis G., Bornman J.F., Nikolopoulos D. (2000). A possible optical role of the bundle sheath extensions of the heterobaric leaves of *Vitis vinifera* and *Quercus coccifera*. Plant Cell Environ..

[41] Geigenberger P. (2003). Response of plant metabolism to too little oxygen. Curr. Opin. Plant Biol..

[42] Méchin V., Thévenot C., Le Guilloux M., Prioul J.L., Damerval C. (2007). Developmental analysis of maize endosperm proteome suggests a pivotal role for pyruvate orthophosphate dikinase. Plant Physiol..

[43] Strasser R.J., Tsimili-Michael M., Srivastava A., Papageorgiou G.C. (2004). Analysis of the chlorophyl *a* fluorescence transient. Chlorophyll a Fluorescence. A signature of Photosynthesis.

[44] Stirbet A. (2011). On the relation between the Kautsky effect (chlorophyll a fluorescence induction) and Photosystem II: Basics and applications of the OJIP fluorescence transient. J. Photochem. Photobiol. B.

[45] Jiang H.X., Chen L.S., Zheng J.G., Han S., Tang N., Smith B.R. (2008). Aluminum-induced effects on Photosystem II photochemistry in Citrus leaves assessed by the chlorophyll a fluorescence transient. Tree Physiol..

[46] Oukarroum A., Schansker G., Strasser R.J. (2009). Drought stress effects on photosystem I content and photosystem II thermotolerance analyzed using Chl a fluorescence kinetics in barley varieties differing in their drought tolerance. Physiol. Plant..

[47] Krüger G.H., Tsimilli-Michael M., Strasser R.J. (1997). Light stress provokes plastic and elastic modifications in structure and function of photosystem II in camellia leaves. Physiol. Plant..

[48] Di Castri F. (1973). Climatographical comparisons between Chile and the western coast of North America. Mediterranean Type Ecosystems.

[49] Flexas J., Diaz-Espejo A., Gago J., Gallé A., Galmés J., Gulías J., Medrano H. (2014). Photosynthetic limitations in Mediterranean plants: A review. Environ. Exp. Bot..

[50] Flexas J., Medrano H. (2002). Drought-inhibition of photosynthesis in C3 plants: Stomatal and non-stomatal limitations revisited. Ann. Bot..

[51] Galmés J., Medrano H., Flexas J. (2007). Photosynthetic limitations in response to water stress and recovery in Mediterranean plants with different growth forms. New Phytol..

[52] Valentini R., Epron D., de Angelis P., Matteucci G., Dreyer E. (1995). In situ estimation of net CO_2_ assimilation, photosynthetic electron flow and photorespiration in Turkey oak (*Q. cerris* L.) leaves: Diurnal cycles under different levels of water supply. Plant Cell Environ..

[53] Niyogi K.K. (2000). Safety valves for photosynthesis. Curr. Opin. Plant Biol..

[54] Li P., Cheng L. (2008). The shaded side of apple fruit becomes more sensitive to photoinhibition with fruit development. Physiol. Plant..

[55] Xu O., Wu J., Cao Y., Yang X., Wang Z., Huang J., Xia G., Zhang O., Hu Y. (2016). Photosynthetic characteristics of leaves and fruits of Hickory (*Carya cathayensis* Sarg.) and Pecan (*Carya illinoensis* K. Koch) during fruit development stages. Trees.

[56] Simkin A.J., Faralli M., Ramamoorthy S., Lawson T. (2020). Photosynthesis in non-foliar tissues: Implications for yield. Plant J..

[57] Papageorgiou G., Demmig-Adams B., Garab G., Adams W. (2014). The non-photochemical quenching of the electronically excited state of chlorophyll a in plants: Definitions, timelines, viewpoints, open questions. Nonphotochemical Quenching and Energy Dissipation in Plants, Algae and Cyanobacteria.

[58] Ogaya R., Peñuelas J. (2003). Comparative seasonal gas exchange and chlorophyll fluorescence of two dominant woody species in a Holm Oak Forest. Flora.

[59] Marques da Silva J. (2007). Chlorophyll fluorescence parameters of three Mediterranean shrubs in a summer-autumn period in central Portugal. Biol. Plant..

[60] Sofo A., Dichio B., Montanaro G., Xiloyannis C. (2009). Photosynthetic performance and light response of two olive cultivars under different water and light regimes. Photosynthetica.

[61] García-Plazaola J.I., Faria T., Abadia J., Chaves M.M., Pereira J.S. (1997). Seasonal changes in xanthophyll composition and photosynthesis of cork oak (*Quercus suber* L.) leaves under Mediterranean climate. J. Exp. Bot..

[62] Baquedano F.J., Castillo F. (2007). Drought tolerance in the mediterranean species *Quercus coccifera*, *Quercus ilex*, *Pinus halepensis*, and *Juniperus phoenicea*. Photosynthetica.

[63] Wittmann C., Aschan G., Pfanz H. (2001). Leaf and twig photosynthesis of young beech (Fagus sylvatica) and aspen (*Populus tremula*) trees grown under different light regime. Basic Appl. Ecol..

[64] Wellburn A.R. (1994). The spectral determination of chlorophylls a and b, as well as total carotenoids, using various solvents with spectrophotometers of different resolution. J. Plant Physiol..

[65] Tsimilli-Michael M., Strasser R.J., Varma A. (2008). In vivo assessment of stress impact on plant’s vitality: Applications in detecting and evaluating the beneficial role of mycorrhization on host plants. Mycorrhiza 3.

[66] Genty B., Briantais J.-M., Baker N.R. (1989). The relationship between the quantum yield of photosynthetic electron transport and quenching of chlorophyll fluorescence. Biochim. Biophys. Acta.

[67] Maxwell K., Johnson G.N. (2000). Chlorophyll fluorescence—A practical guide. J. Exp. Bot..

[68] IMAGING-PAM M-Series Chlorophyll Fluorometer (2014). Instrument Description and Information for Users.

[69] Zeliou K., Manetas Y., Petropoulou Y. (2009). Transient winter leaf reddening in *Cistus creticus* characterizes weak (stress-sensitive) individuals, yet anthocyanins cannot alleviate the adverse effects on photosynthesis. J. Exp. Bot..

[70] Tseliou E., Chondrogiannis C., Kalachanis D., Goudoudaki S., Manoussopoulos Y., Grammatikopoulos G. (2021). Integration of biophysical photosynthetic parameters into one photochemical index for early detection of Tobacco Mosaic Virus infection in pepper plants. J. Plant Physiol..

